# Estimation of hospital visits for respiratory diseases attributable to PM_10_ from vegetation fire smoke and health impacts of regulatory intervention in Upper Northern Thailand

**DOI:** 10.1038/s41598-022-23388-2

**Published:** 2022-11-02

**Authors:** Athicha Uttajug, Kayo Ueda, Akiko Honda, Hirohisa Takano

**Affiliations:** 1 Department of Environmental Engineering, Graduate School of Engineering, Kyoto University, Kyoto, Japan; 2 Department of Hygiene, Graduate School of Medicine, Hokkaido University, Sapporo, Japan; 3 Graduate School of Global Environmental Studies, Kyoto University, Kyoto, Japan

**Keywords:** Public health, Environmental impact

## Abstract

The air quality in Upper Northern Thailand (UNT) deteriorates during seasonal vegetation fire events, causing adverse effects especially on respiratory health outcomes. This study aimed to quantitatively estimate respiratory morbidity from vegetation fire smoke exposure, and to assess the impact of a burning ban enforced in 2016 on morbidity burden in UNT. We computed daily population exposure to fire-originated PM_10_ and estimated its health burden during a 5-year period from 2014 to 2018 using daily fire-originated PM_10_ concentration and the concentration–response function for short-term exposure to PM_10_ from vegetation fire smoke and respiratory morbidity. In subgroups classified as children and older adults, the health burden of respiratory morbidity was estimated using specific effect coefficients from previous studies conducted in UNT. Finally, we compared the health burden of respiratory morbidity before and after burning ban enforcement. Approximately 130,000 hospital visits for respiratory diseases were estimated to be attributable to fire-originated PM_10_ in UNT from 2014 to 2018. This estimation accounted for 1.3% of total hospital visits for respiratory diseases during the 5-year period, and 20% of those during burning events. Age-specific estimates revealed a larger impact of PM_10_ in the older adult group. The number of hospital visits for respiratory diseases attributable to fire-originated PM_10_ decreased from 1.8% to 0.5% after the burning ban policy was implemented in the area. Our findings suggest that PM_10_ released from vegetation fires is a health burden in UNT. The prohibition of the burning using regulatory measure had a positive impact on respiratory morbidity in this area.

## Introduction

Vegetation fire events, including forest fires, grass fires, and open field burning for agricultural practices and plantation management, are significant sources of air pollution in many Southeast Asian (SEA) countries^1,2^. Due to proximity to the equatorial Pacific Ocean, smoke haze events in these countries are worsened by drought conditions during the El Nino phenomenon^3^. Smoke haze events have frequently observed in the maritime SEA region, including Indonesia, Malaysia, and Singapore since 1997^4^. Recently, Mainland Southeast Asia (MSEA), which covers the continental land area (i.e., Vietnam, Laos, Cambodia, Myanmar, and upper northern Thailand), has also suffered from local and transboundary air pollution due to vegetation fires^5^.

In Thailand, smoke haze from vegetation fires is a common occurrence across Upper Northern Thailand (UNT) during dry seasons (January to April). Fires are mostly used to clear vegetation to collect non-timber forest products (i.e., mushroom and bamboo shoot)^6^. Forests represent the predominant burned area in UNT^7^. In order to address this problem, the government has implemented several control measures in UNT since 2004. However, seasonal smoke haze continues affect the area. In 2016, a prohibition of burning using National Reserved Forest Act was enforced with the strict penalties^8^. A previous study reported that the ban led to decreased burning activities, fewer satellite-fire hotspots, and lower PM_10_ concentrations in the area^9^.

Previous epidemiological studies have shown that exposure to air pollution emitted from vegetation fires is associated with respiratory health outcomes^10–25^. Despite the growing epidemiological evidence, few studies estimated the health burden of vegetation fire smoke exposure. One study estimated that more than 300,000 premature deaths are attributable to exposure to PM emitted from vegetation fires, with the highest number of deaths occurring in sub-Saharan Africa and Southeast Asia^26^. In Southeast Asia, some studies assessed the health burden of vegetation fire smoke in the Maritime region^27–32^.

To date, no study has quantified the health burden of air pollutants from vegetation fires in MSEA, where the sources of vegetation fires differ from other areas (i.e., peatland fire in Maritime SEA). The present study aimed to quantitatively estimate the number of hospital visits for respiratory diseases attributable to short-term exposure to PM_10_ from vegetation fires in UNT.

## Results

### Hospital visits for respiratory diseases in UNT

From 2014 to 2018, there were roughly 2 million hospital visits for respiratory diseases annually (Table 1). Nearly half of these visits were made by children, and 15% by older adults. The daily average of total hospital visits for respiratory diseases decreased after the enforcement of the 2016 burning ban for a 5-year period and burning days.

**Table 1:** Summary of hospital visits for respiratory diseases in UNT during 2014–2018.

	2014	2015	2016	2017	2018	2014–2018
**5-years period**						
Total count	1,834,682	2,124,322	2,377,088	2,184,780	1,925,402	10,446,274
Daily average	689	728	807	743	690	731
Children (%)	49.2	50.4	50.3	48.6	47.0	49.2
Older adults (%)	14.5	14.1	14.7	16.4	17.5	15.4
**Burning days**						
Total count	173,396	158,019	207,756	51,524	61,717	652,412
Daily average	788	802	802	661	726	756
Children (%)	43.9	45.3	47.2	44.0	41.8	45.1
Older adults (%)	15.3	14.9	15.6	17.8	18.7	15.8

### Fire-originated and background PM_10_

**Figure 1:**
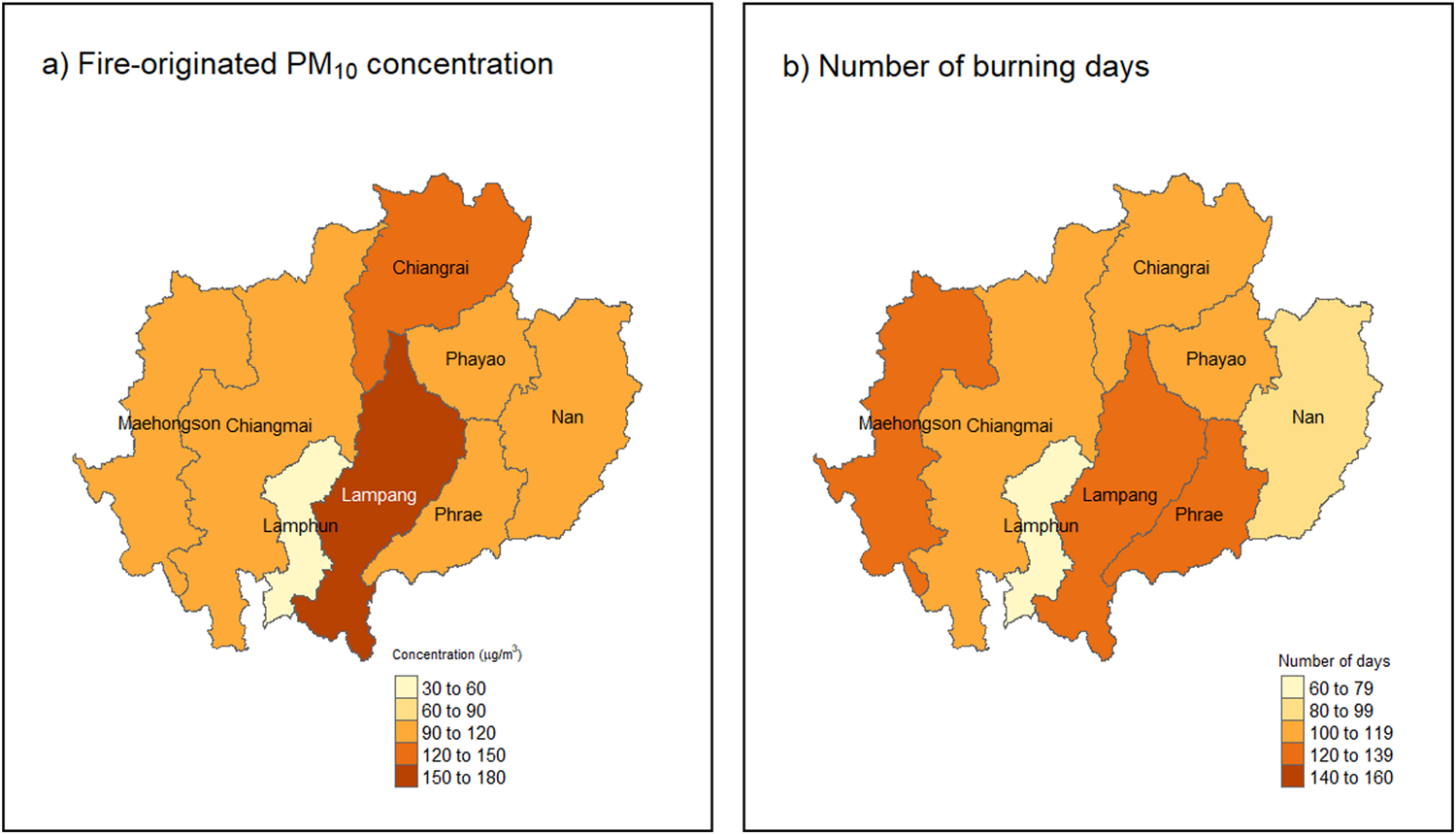
Average fire-originated PM_10_ concentration (μg/m^3^) and total number of burning days. Map was generated using the package “*raster*”^46^ and “*tmap*”^47^ of R (version 4.1.3, The R Foundation for Statistical Computing, Vienna, Austria).

**Figure 2:**
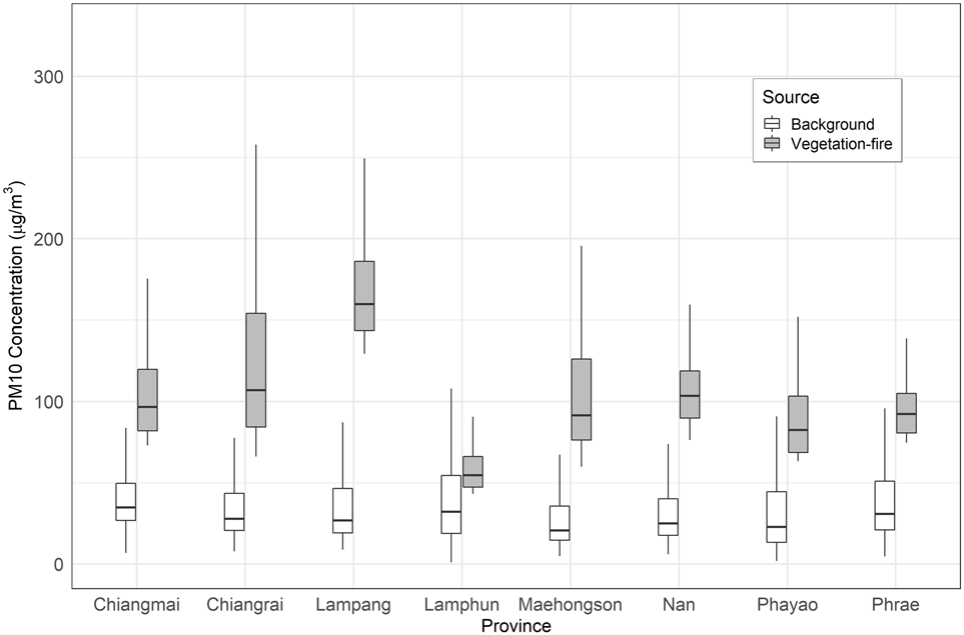
Boxplot of background and vegetation-fire-originated PM_10_ concentration.

Daily average fire-originated PM_10_ concentrations ranged from 58.7 to 171.9 μg/m^3^ across the eight provinces in UNT (mean: 106.5 μg/m^3^), and the numbers of burning days ranged from 64 days (Lamphun) to 122 days (Lampang) (Fig. 1). The daily average background concentration ranged from 21.3 μg/m^3^ to 30.4 μg/m^3^ (mean: 23.2 μg/m^3^, which was lower than almost one fifth of the average fire-originated PM_10_ concentration) (Fig. 2). The average fire-originated PM_10_ concentration and the average number of burning days decreased after burning ban enforcement from 114.6 to 94.5 μg/m^3^ and 233 to 82 days, respectively (Table 2).

**Table 2:** Fire-originated PM_10_ concentration and estimated number of hospital visits attributable to fire-originated PM_10_ during 2014–2018 for eight provinces.

Variables	Study period (year)						Burning ban enforcement (annual average)	
	2014	2015	2016	2017	2018	2014–2018	Before (2014–2016)	After (2017–2018)
**Environmental variables**								
Total number of burning days	244	197	259	78	85	863	233.3	81.5
Average population weighted fire-originated PM_10_	115	120.7	108.2	98.5	90.4	106.6	114.6	94.5
**Number of attributable cases and uncertainty range (thousand)**								
All ages	36.6	35.9	40.5	9.4	10.5	132.9	37.6	9.9
	(23.2–48.6)	(22.9–47.3)	(25.6–54.0)	(5.9–12.5)	(6.6–14.0)	(84.2–176.5)	(24.0–50.0)	(6.3–13.3)
Children	7.7	7.9	9.3	1.9	2.0	28.9	8.3	2.0
	(0.9–13.9)	(0.9–14.1)	(1.1–16.6)	(0.2–3.4)	(0.2–3.7)	(3.4–51.7)	(1.0–14.9)	(0.2–3.6)
Older adults	5.9	5.6	6.7	1.8	2.1	22.2	6.1	2.0
	(2.1–9.0)	(2.0–8.4)	(2.4–10.2)	(0.6–2.8)	(0.7–3.2)	(8.1 -33.7)	(2.2–9.2)	(0.7–3.0)
**Proportion of attributable cases for 5-years period (%)**								
All ages	2.0	1.7	1.7	0.4	0.5	1.3	1.8	0.5
Children	0.9	0.7	0.8	0.2	0.2	0.6	0.8	0.2
Older adults	2.2	1.9	1.9	0.5	0.6	1.4	2.0	0.6
**Proportion of attributable cases for burning days (%)**								
All ages	21.1	22.7	19.5	18.2	17.0	20.3	21.0	17.6
Children	10.2	11.1	9.5	8.4	7.9	9.4	10.2	8.2
Older adults	22.4	23.9	20.6	20.1	18.4	21.1	22.1	19.2

### Hospital visits for respiratory diseases attributed to fire-originated PM_10_

**Figure 3:**
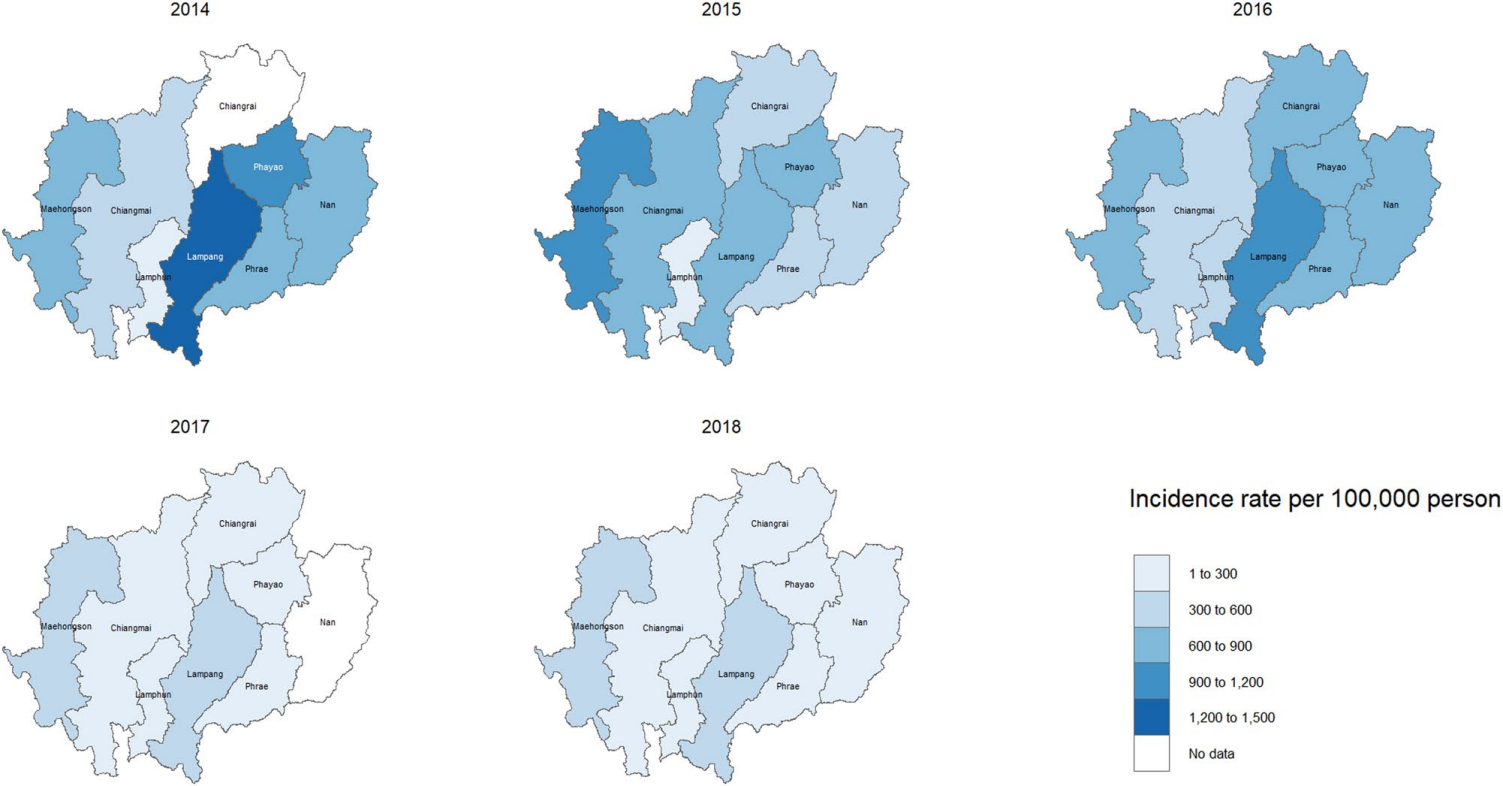
Incidence rates of annual total hospital visits for respiratory diseases attributable to fire-originated PM_10_ for all ages during 2014–2018 by province. Map was generated using the package “*raster*”^46^ and “*tmap*”^47^ of R (version 4.1.3, The R Foundation for Statistical Computing, Vienna, Austria).

The estimated number of hospital visits for respiratory diseases attributable to fire-originated PM_10_ for all ages throughout the study period was 132,923 (Table 2). One third of these hospital visits were made by vulnerable groups (children: 28,937 visits, older adults: 22,207 visits). This estimation of total attributable cases accounted for approximately 1.3% of total hospital visits for respiratory diseases during the 5-year period and 20.3% during burning days. The proportion of hospital visits attributable to fire-originated PM_10_ was greater in older adults (1.4%) than in children (0.6%). The incidence rate of attributable cases for all ages and in vulnerable groups by province-year are shown in Fig. 3 and Supplementary Figures (S2 and S3). We observed the largest incidence rates in Lampang (4,244 cases per 100,000 persons for 5-year period) (Fig. 3).

### Impact of a vegetations fire events ban on hospital visits for respiratory diseases

After burning ban enforcement in 2016, the annual average number of hospital visits for respiratory diseases attributable to fire-originated PM_10_ decreased from 37,682 to 9944 (approximately 70% reduction from the pre-intervention period) (Table 2), which is consistent with the decrease in the total number of hospital visits during burning days (Table 1). The proportion of total attributable cases for 5-year period decreased by 1.3% (from 1.8% to 0.5%). Simultaneously, the proportion of attributable cases also decreased by 0.6% and 1.4% for children and older adults, respectively, after burning ban enforcement in the area. The decrease in the proportion of attributable cases during burning days (− 3.4%) was greater than that during the 5-year period (− 1.3%) for all ages after ban enforcement.

### Sensitivity results

The sensitivity analysis using a lower cut-off concentration (50 μg/m^3^) of fire-originated PM_10_ revealed that 2.4% and 12.6% of total hospital visits for respiratory diseases were attributable to fire-originated PM_10_ during the 5-year period and burning days, respectively (Table S1).

## Discussion

The population-weighted daily average concentration of PM_10_ from vegetation fires across UNT during 2014–2018 was 106.5 μg/m^3^ (range: 58.7–171.9 μg/m^3^). In general, fire-originated PM_10_ concentrations was lower after burning ban enforcement in 2016.

Despite the growing concern about air pollution caused by vegetation fire events, its far-reaching health effects are often ignored. The present study showed that exposure to particles emitted from vegetation fire events throughout UNT poses health risks, such as increased respiratory morbidity, with 132,923 hospital visits (1.3% of total) being attributed to fire-originated PM_10_ for all ages. Moreover, approximately one-third of these visits occurred in vulnerable groups. We found that Lampang, which was the province with the highest PM_10_ concentration from fire events, had the highest incidence rate of attributable cases among UNT region. The number of hospital visits for respiratory diseases attributable to PM_10_ decreased after burning ban enforcement.

Only a few studies have estimated the health burden of exposure to air pollution from vegetation fire events, particularly in terms of morbidity. Previously studies mainly addressed mortality on a global scale or in the equatorial Southeast Asian region^26–29,31–35^. Some studies used morbidity as a health outcome, such as a study in Australia which examined hospitalization for cardiovascular disease and asthma^36^, and another that targeted respiratory diseases in the United States^37^. While the impact of long-term exposure to particles from all sources in Thailand has been reported^38^, no study has estimated morbidity impacts of short-term exposure to particles emitted from vegetation fire events in MSEA. The study estimated the number of health burden attributable to fire-originated PM_10_ is needed because air pollution from these events has continuously affected people in UNT and it may be useful for further policy making.

Quantifying the health burden of air pollution exposure due to vegetation burning may be useful from a policy-making perspective. We observed decreases in fire-originated PM_10_ concentration, number of burning days, and number of hospital visits for respiratory diseases attributable to PM_10_ after burning ban enforcement. These findings are consistent with previous reports that PM_10_ concentrations in the area have decreased since the enforcement of the burning ban policy^9^. While the policy may have helped reduce toxic components of particles emitted from burning activities, such as carbonaceous aerosols (black and organic carbon), Polycyclic Aromatic Hydrocarbons (PAHs), and levoglucosan^39^, it does not appear to offer sustainable measures against smoke haze events. In fact, we observed increases in fire-originated PM_10_ concentration as well as the number of hospital visits attributable to PM_10_ in 2018 (i.e., after ban enforcement).

In addition to the policy, global climate factors may have influenced PM_10_ emission from vegetation fires. The strong El Nino phenomenon was observed during 2015–2016, resulting in dry conditions, followed by La Nina events (i.e., wet climate) in 2017^40^. During the study period, we estimated the highest number of hospital visits for respiratory diseases attributable to fire-originated PM_10_ to be approximately 40,000 during the time of strong El Nino (2015–2016). A previous study also estimated a high global health burden attributable to particles released from burning sources due to the influence of El Nino^41^.

There are some limitations to this study. In exposure assessment, we estimated the health burden of PM_10_ exposure using PM_10_ concentrations derived from ambient air pollution monitoring data, which may not have accurately reflected actual individual exposure. The inaccurate number of hospital visits for respiratory diseases attributable to fire-originated PM_10_ may be caused from several stages of health burden estimation (i.e., exposure assessment and applying of concentration response function). To identify burning days, we used a cut-point reported in a previous study for the occurrence of intensive fires. PM_10_ concentrations on the remaining days (i.e., non-burning days) were averaged as the background concentration, but small burning events might have occurred during non-burning days, contributing to the estimated background concentration. However, the background PM_10_ concentration did not differ from PM_10_ concentrations reported for non-burning months (May-December) in a previous study^20^.

According to the WHO guideline, the concentration of daily PM_10_ should not exceeded 50 μg/m^3^. We thus performed a sensitivity analysis by changing the cut-point from 100 μg/m^3^ to 50 μg/m^3^ in order to capture more burning events, and to lower the average fire-originated PM_10_ concentration as compared to the principal analysis. The proportions of estimated hospital visits during the 5-year period did not significantly differ between principal and sensitivity analyses, but the proportion during burning days was smaller when using the WHO guideline concentration. These results suggest that using the guideline concentration, which has been set based on ambient air particles, may lead to underestimation.

Despite these limitations, the present study has the following strengths. We used effect coefficients obtained from epidemiological studies that conducted with the same health outcomes in UNT. This might have helped reduce the uncertainty of health burden estimation attributable to fire-originated PM_10_ because the same factors were considered such as health care system, vegetation fire particle compositions, and behavioral responses to the smoke haze of people in this area. Another strength is that we estimated the number of hospital visits for respiratory diseases attributable to fire-originated PM_10_ in vulnerable groups. We found a larger impact of short-term exposure to fire-originated PM_10_ among older adults. With the increasing aging population, this study highlights the need to address the effect of burning events on the health of older people. Our findings may help prepare for and implement preventive measures against smoke haze risk in vulnerable populations.

## Conclusion

Short-term exposure to PM_10_ emitted from vegetation fire events was associated with approximately 130,000 hospital visits for respiratory diseases in UNT during a 5-year period. In particular, the estimated number of hospital visits attributable to PM_10_ was high among older adults. These findings may be useful for further advancing policy-making regarding haze control and overall health and socioeconomic consequences. Moreover, our results suggest that regulatory actions on vegetation fire events had a positive impact on hospital visits for respiratory diseases in UNT.

## Methods

### Identification of burning days and estimation of fire-originated PM_10_

Hourly data of PM_10_ concentration were obtained from 14 air quality monitoring stations in eight provinces of UNT (Chiangrai, Chiangmai, Lampang, Lamphun, Maehongson, Nan, Phayao, and Phrea), provided by the Pollution Control Department of Thailand, from January 2014 to December 2018. Daily averages were estimated when 75% daily records were available (at least 18 valid hourly records). Initially, we calculated the population weighted PM_10_ concentration to refine exposure estimation, as shown in the following equation Eq. (1).

1 $$Population\; weighted\; PM_{10} = \sum \frac{{C_{i} \times P_{i} }}{{P_{tot} }}$$

where ${C}_{i}$ is the PM_10_ concentration, ${P}_{i}$ is the population of district i (in each province), and ${P}_{tot}$ is the total population of each province^42^. The population data of each district was retrieved from Gridded Population of the World, version 4^43^.

We estimated the fire-originated PM_10_ concentration by subtracting background PM_10_ concentration from daily average PM_10_ concentration on burning days:

2 $$PM_{{10 \left( {{\text{fire}} - {\text{sourced}}} \right)}} = PM_{{10 \left( {daily} \right)}} - PM_{{10 \left( {background} \right)}}$$

In this step, we identified burning days based on criteria described in a previous study^25^. Briefly, a burning day was identified when the number of satellite fire hotspots exceeded the 90^th^ percentile of the daily distribution in the entire region (10 counts) and the daily PM_10_ concentration in each province was greater than 100 μg/m^3^. The fire hotspot data was obtained from the National Aeronautics and Space Administration Land, Atmosphere Near real-time Capability for Earth observing system (NASA-LANCE) for Fire Information for Resource Management System (FIRMS)^44^. In this study, individual fire hotpots with lower confidence values (< 20%) were excluded from the analyses to obtain the precise burning point. Moreover, the cut-point of PM_10_ concentration (> 100 μg/m^3^) was selected based on the previous study findings^23^. The background concentration was derived by averaging the estimated daily PM_10_ concentrations after adjusting for day-of-week (DOW) and seasonal patterns on non-burning days (fire hotspot = 0). Adjustments for temporal trends were performed by using a natural cubic spline of time with 5 degrees of freedom (df) per year and DOW (Supplementary Figure S1). We changed df of time from 4 to 6 to check the robustness. The minimal adequate model was chosen by the Akaike Information Criterion and ANOVA tests.

### Concentration–response function and morbidity burden assessment

We estimated the number of hospital visits for respiratory diseases attributable to PM_10_ for all ages and vulnerable groups (children and older adults) in each province. Data were obtained from the Ministry of Public Health of Thailand for each province and included demographic information (sex and age), date of visit, and International Classification of Disease codes for diagnosis (ICD10: J00-J99). We estimated the number of hospital visits for respiratory diseases attributable to fire-originated PM_10_ between January 2014 and December 2018 using methods described previously^33^.

3 $$RR = {\text{exp}}\left( {\beta \times \left( {PM_{{10 \left( {{\text{fire}} - {\text{sourced}}} \right)}} } \right)} \right)$$

where RR is the relative risk of daily average fire-originated PM_10_ concentration on burning days for each province. Among few epidemiological studies conducted in this region^20,25,45^, we used the coefficient $\beta$ derived from Mueller and colleagues because the subjects included in this study are truly represented the population in this area^20^. Specifically, Mueller and colleagues reported the risk of hospital visits for respiratory diseases to be 1.020 (95% CI: 1.012, 1.028) per 10 μg/m^3^ increase in PM_10_ for all ages. Accordingly, $\beta$ was calculated as In(1.020) per 10 μg/m^3^. The same estimation method was used to calculate the health burden in vulnerable groups, that is, children under age 15 (1.009 (95% CI: 1.001, 1.017) for the risk of hospital visits for respiratory diseases)^25^ and older adults aged ≥ 65 years (1.021 (95% CI: 1.007, 1.035) for the risk of outpatient visits for chronic lower respiratory diseases)^20^.

The number of daily hospital visits for respiratory diseases attributable to fire-originated PM_10_ in each province was calculated using the following equation:

4 $$Attributable\; cases = HV \times \frac{{\left( {RR - 1} \right)}}{RR}$$

where HV is the daily number of hospital visits for respiratory diseases. The fraction of risk function (RR-1)/RR is defined as the population attributable fraction (PAF), which measures the disease burden attributable to a risk from exposure to fire-originated PM_10_. We summed number of the attributable cases by year and province. The proportion of attributable cases was estimated from the number of attributable cases divided by the total number of cases in each year and age group. We also calculated the incidence rate of the attributable cases for each province using the population data in 2015 derived from the National Statistical Office of Thailand.

Finally, we performed a sensitivity analysis to address the uncertainty of health burden estimation. We estimated the health burden by changing the cut-point for burning days from 100 μg/m^3^ to 50 μg/m^3^ according to the WHO guideline for daily PM_10_ concentration in order to capture lower concentration exposure that could affect health outcomes.

### Ethical considerations

This study was exempt from ethical approval by the Ethics Committee of Kyoto University Graduate School of Engineering (No. 201904), since only secondary and aggregated data were used in the analyses.

## Supplementary Information

Supplementary Information.

## Data Availability

The data that support the findings of this study are available from Permanent Secretary Ministry of Public Health Thailand but restrictions apply to the availability of these data, which were used under license for the current study, and so are not publicly available. Data are however available from the authors upon reasonable request and with permission of Permanent Secretary Ministry of Public Health Thailand.
