# Supplementary material for: Estimation of hospital visits for respiratory diseases attributable to PM10 from vegetation fire smoke and health impacts of regulatory intervention in Upper Northern Thailand
Source: Sci Rep. 2022 Nov 2;12:18515. doi: 10.1038/s41598-022-23388-2 (PMC9630449; doi:10.1038/s41598-022-23388-2)
Supplement: Supplementary file 1 — Supplementary Information. [file 41598_2022_23388_MOESM1_ESM.docx]

**Supplementary materials**

**Title: Estimation of hospital visits for respiratory diseases attributable to PM_10_ from vegetation fire smoke and health impacts of regulatory intervention in Upper Northern Thailand**

Athicha Uttajug*^1,2^, Kayo Ueda^2^, Akiko Honda^1,3^, Hirohisa Takano^1,3^

***Affiliations***

^1^ Department of Environmental Engineering, Graduate School of Engineering, Kyoto University, Kyoto, Japan.

^2^ Department of Hygiene, Graduate School of Medicine, Hokkaido University, Hokkaido, Japan.

^3^ Graduate School of Global Environmental Studies, Kyoto University, Kyoto, Japan.

Table S1. Sensitivity analysis using a cut-point of 50 μg/m^3^ rather than 100 μg/m^3^ for identifying burning days

| Year | Number of burning days | Population-weighted fire-originated PM_10_ (μg/m^3^) | Number of hospital visits attributable to fire-originated PM_10_ (uncertainty range) | % attributable | |
| --- | --- | --- | --- | --- | --- |
|  |  |  |  | 5-years period | Burning day |
| 2014-2018 | 2,643 | 67.2 | 254,897 (159,435, 342,802) | 2.4 | 12.6 |
| 2014 | 551 | 76.5 | 54,281(34,102, 72,686) | 2.9 | 15.0 |
| 2015 | 523 | 74.6 | 57,350 (36,119, 76,637) | 2.7 | 14.1 |
| 2016 | 645 | 75.9 | 70,549 (44,177, 94,765) | 2.9 | 14.1 |
| 2017 | 470 | 52.7 | 35,842 (22,182, 48,691) | 1.7 | 10.0 |
| 2018 | 454 | 56.5 | 36,876 (22,855, 50,023) | 1.9 | 11.3 |


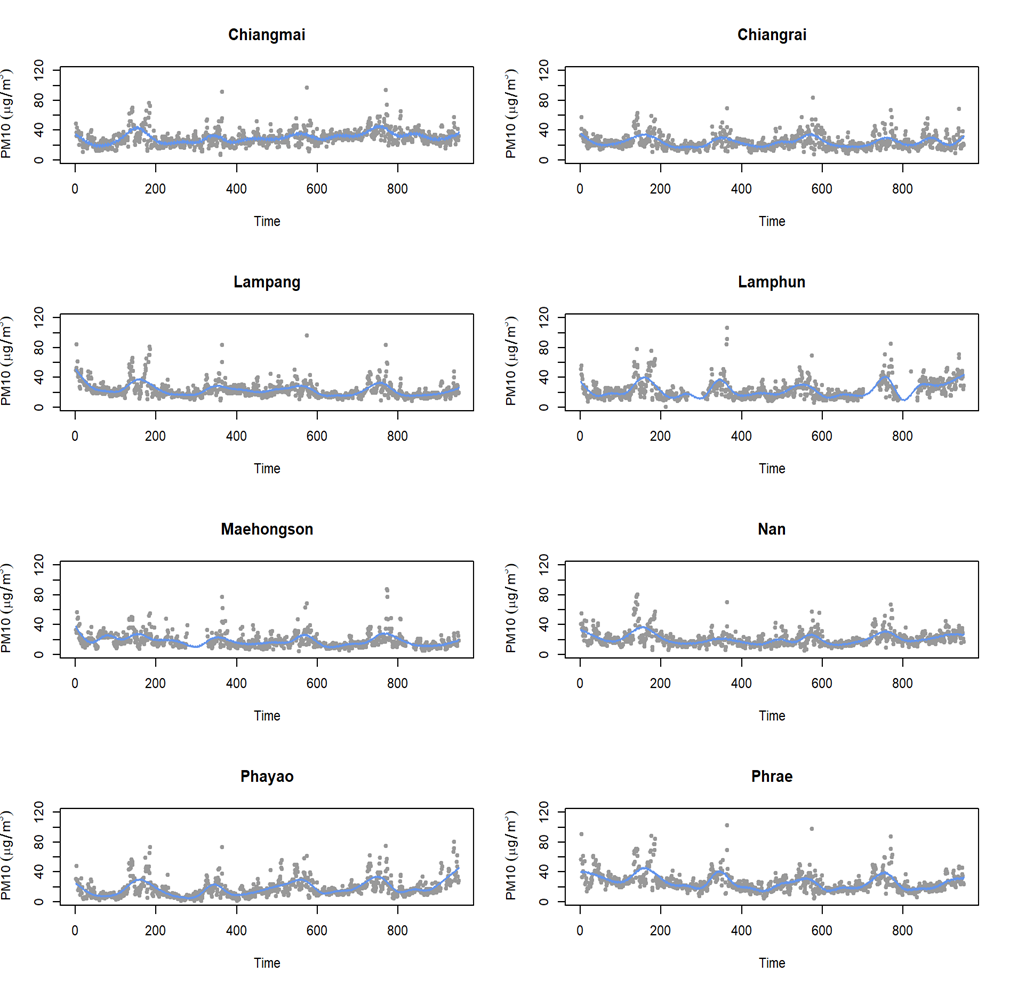


Figure S1. Time-series of the original PM_10_ concentration from ground monitoring station in eight provinces in Upper Northern Thailand during non-burning days (gray points) and the mean of estimated PM_10_ concentration after adjusting for seasonality and day of week (blue line).


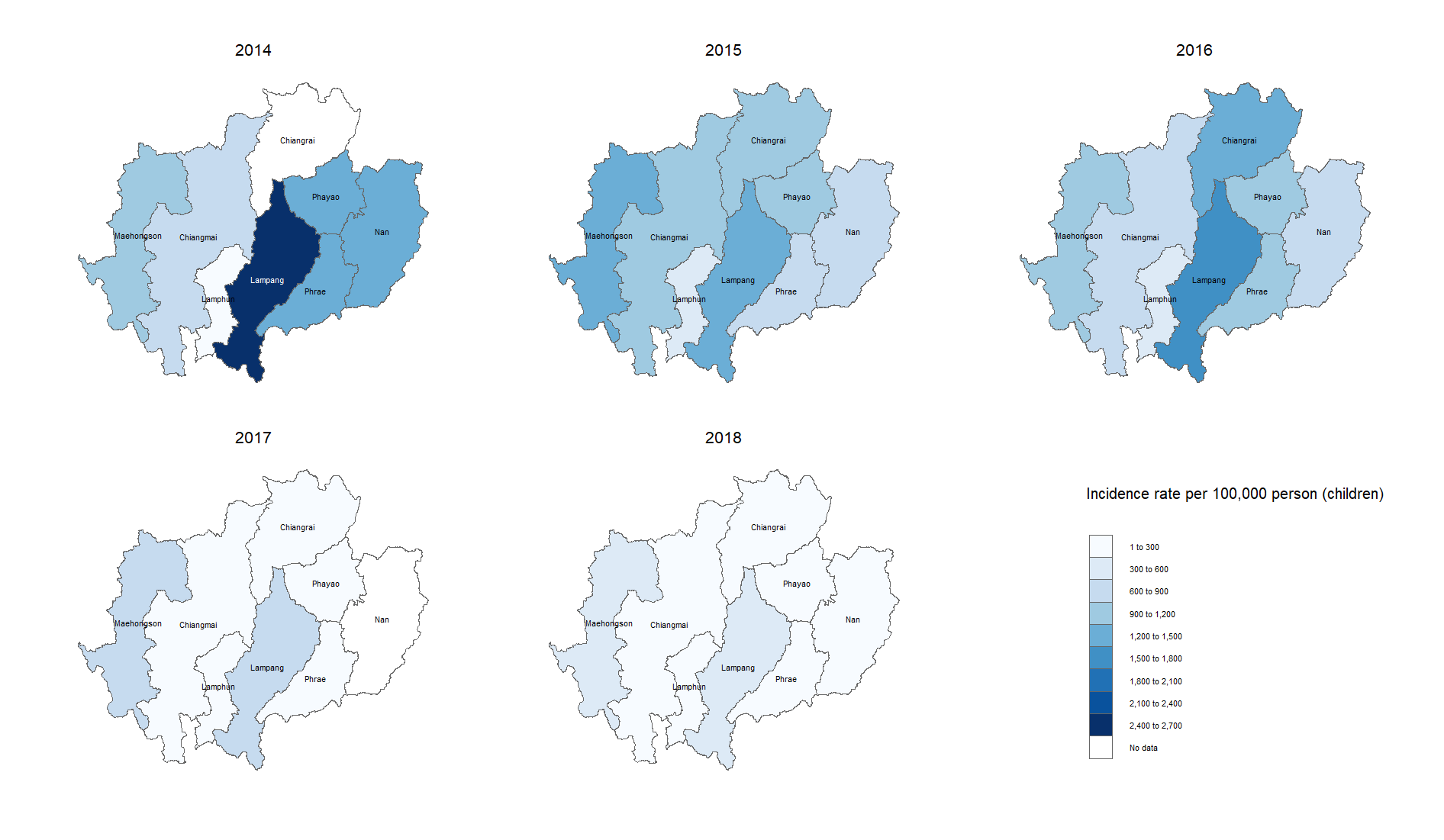


Figure S2. Incidence rate of hospital visits for respiratory diseases attributable to fire-originated PM_10_ among children during 2014-2018 by province. Map was generated using the package “*raster*”^1^ and “*tmap*”^2^ of R (version 4.1.3, The R Foundation for Statistical Computing, Vienna, Austria).


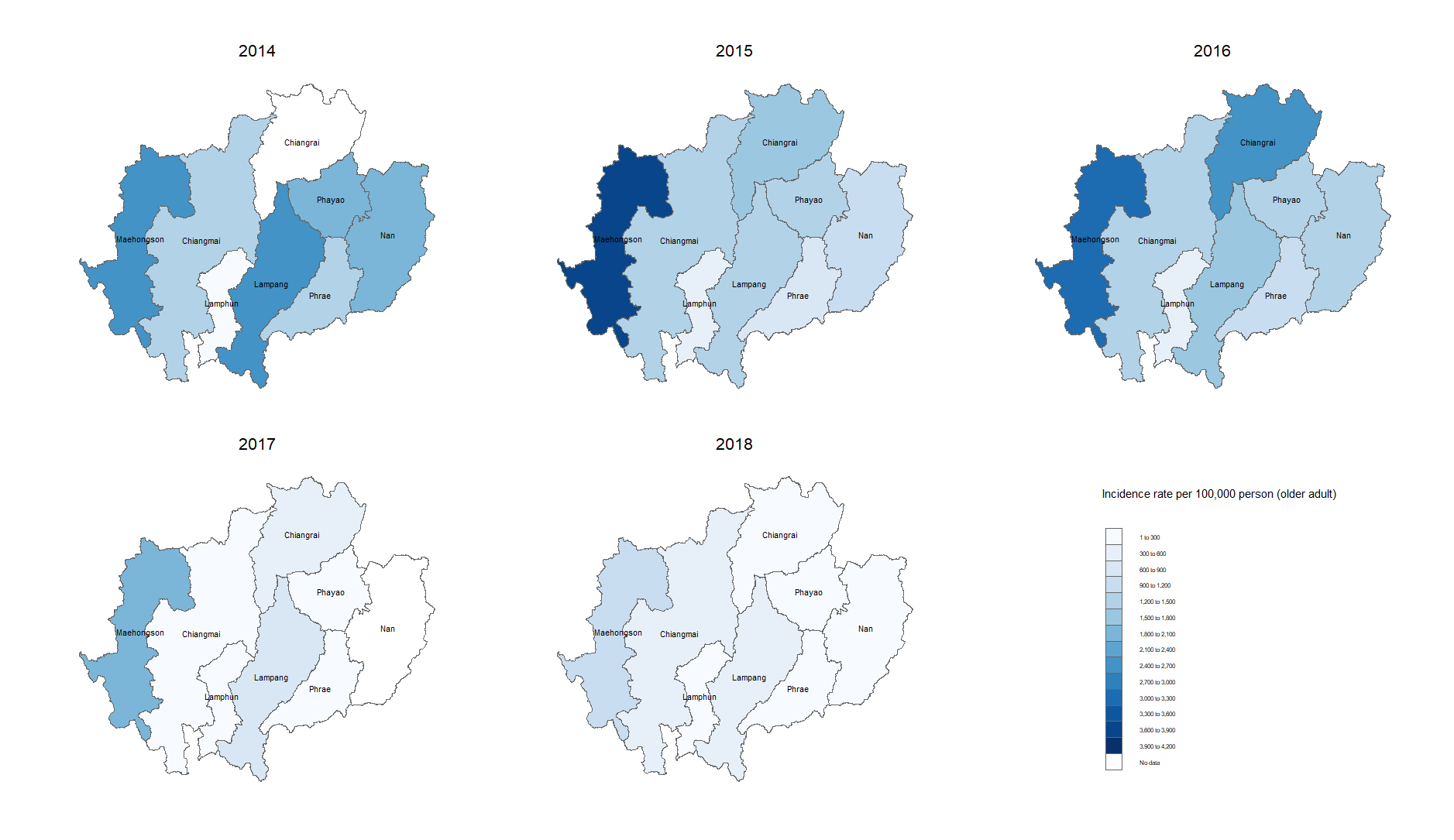


Figure S3. Incidence rate of hospital visits for respiratory diseases attributable to fire-originated PM_10_ among older adults during 2014-2018 by province. Map was generated using the package “*raster*”^1^ and “*tmap*”^2^ of R (version 4.1.3, The R Foundation for Statistical Computing, Vienna, Austria).

**Reference**

1 Robert, J., Hijmans, & Jacob, v., Etten. *raster: Geographic analysis and modeling with raster data*, http://CRAN.R-project.org/package=raster (2012).

2 Tennekes, M. tmap: Thematic Maps in R. *Journal of Statistical Software* **84**, doi:10.18637/jss.v084.i06 (2018).
